# ARID2, a Rare Cause of Coffin–Siris Syndrome: A Clinical Description of Two Cases

**DOI:** 10.3389/fped.2022.911954

**Published:** 2022-06-23

**Authors:** Xiaoyan Wang, Haiying Wu, Hui Sun, Lili Wang, Linqi Chen

**Affiliations:** Department of Endocrinology, Children’s Hospital of Soochow University, Suzhou, China

**Keywords:** Coffin-Siris syndrome, *ARID2*, exome sequenci, short stature, SWI/SNF (BAF) complex

## Abstract

**Background:**

Coffin-Siris syndrome (CSS) is a multiple congenital anomaly syndrome characterized by coarse facial features, sparse scalp hair, hypertrichosis, and hypo/aplastic digital nails and phalanges. Mutations in the BAF (SWI/SNF)-complex subunits (SMARCE1, SMARCB1, SMARCA4, SMARCA2, ARID1B, and ARID1A) have been shown to cause CSS. People diagnosed with BAF pathway related diseases are increasing, and *ARID2* (NM_152641.4) is the least common of these genes. Mutations in the ARID2 gene is the cause for Coffin-Siris syndrome 6 (CSS6). By now only 16 individuals with CSS have been reported to have pathogenic variants in *ARID2*.

**Case Presentation:**

In this article, we introduced two individuals with clinical features consistent with CSS6 (Coffin-Siris syndrome 6). This article increases the number of reported cases, provides better phenotypic information for this rare syndrome, and allows everyone to better understand the disease.

**Conclusion:**

Our observations indicate that *ARID2* mutations could have variable phenotypes, even in patients from the same family.

## Background

Coffin-Siris Syndrome (CSS, OMIM 135900) is a multiple congenital anomaly syndrome. Current studies have proved that the disease is caused by mutations in human BRG- or HRBM-associated factor (BAF) pathway (1). Within this family of genes, pathogenic variants have been found in *ARID1B* (the most common), *ARID1A*, *SMARCB1, SMARCA4*, and *SMARCE1* in individuals with clinical phenotypes consistent with CSS. More recently, pathogenic alterations of the *ARID2* gene were also observed in probands with neurodevelopmental delay and CSS6 characteristics (2). In general, patients with pathogenic variants of *ARID2* tend to share the core features, such as developmental and growth delay, coarsening of the face, intelligence retardation, and abnormal behavior. Intelligence abnormalities are mainly mild to moderate. Atypical facial features primarily involve rough facial features, such as a protruding forehead, short-broad nose with anteverted nostril, low set and posteriorly rotated ear, a large mouth, thick upper lips, and hypoplasia of the nails or phalanges, typically of the 5th digits (3). To date, only 16 individuals with CSS have been reported to have pathogenic variants in *ARID2*. There are no reports of CSS6 cases in China. Herein, we report two cases of *ARID2* gene mutation (2–7). The medical records of two Chinese patients with CSS6 from the inpatient clinics of the Children’s Hospital of Soochow University in May 2019 were analyzed. Clinical data and results of genetic analysis are reported as follows.

## Materials and Methods

Informed consent was obtained from all individual participants included in the study. This study was approved by the Children’s Hospital of Soochow University.

Whole-exome sequencing (WES) analysis were used to analyze the samples from patients. The mean vertical coverage was 325.14X, and the horizontal coverage was 99.99%.

Genomic DNA was extracted from peripheral blood leukocytes following the manufacturer’s protocols. DNA samples from the 2 cases were fragmented using a Scientz08-III automated sonicator. Massively parallel sequencing was performed using Illumina MiSeq platform according to the standard manual. The raw data were filtered and aligned against the human reference genome (hg19). The single-nucleotide polymorphisms (SNPs) were called by using the GATK software. Variants were annotated using ANNOVAR 3. Effects of single-nucleotide variants (SNVs) were predicted by SIFT, Polyphen-2, and Mutation Taster programs. The detected pathogenicity variants was evaluated according to the recommendations of the ACMG for variant classification and reporting. The associated phenotypic features of candidate genes were analyzed against the patient’s phenotype. Sanger sequencing was used to determine the genotypes of affected and unaffected family members at candidate loci.

### Case Presentation

#### Case 1

**FIGURE 1:**
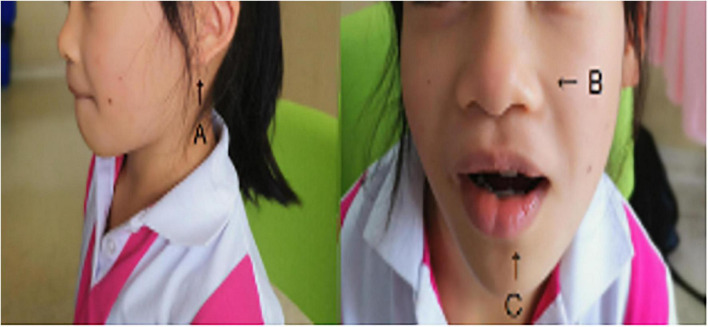
The patient has coarse facial features, low-set and posteriorly rotated ears **(A)**, a flat, upturned nose **(B)**, sparse bitemporal scalp hair, thick eyebrows, and a wide mouth with thick lips **(C)**.

The proband (P1) was born at term weighing 2.6 kg to a non-consanguineous couple. She had no obvious feeding difficulties in infancy. Developmentally, she exhibited global developmental delay, achieving raising head at 7 months, sitting at 9 months, and walking at 18 months. She attended primary school at 7 years and 8 months old with poor academic performance. At present, she is 115 cm tall (< 2 *SD*), weighs 19.5 kg, and has a special face with dysmorphic features (including thick upper lips, micrognathia, and low-set ears). Furthermore, her toenails were noted to be hyperplastic (Figure 1 and Table 1).

**TABLE 1:** Summary of the features of Coffin-Siris syndrome with *ARID2* mutation reported in the literature.

	Variant	*De* *novo*	Facial features	Intellectual disability	Behavior abnormality	Short stature	Birth weight	Age acquired walk (months)	Age acquired first word (months)	GERD	Hypotonia	References #
1	Val846Leufs * 3	+	Micrognathia, posteriorly rotated ears, epicanthal folds, down slanting palpebral fissures, highly arched palate	+	ADHD, anxiety	<5%	5%	24	12	–		(3)
2	Leu343*	UR	Micrognathia, posteriorly rotated ears, epicanthal folds, down slanting palpebral fissures, highly arched palate	+	ADHD, aggressive	25%	25–50%	24	NR	–	+	(3)
3	His1481Ilefs * 4	+	Micrognathia, posteriorly rotated ears, epicanthal folds, down slanting palpebral fissures, highly arched palate	+	Affinity water, sensitive	10%	25%	20	18	+	+	(3)
4	Q1440*	+	Micrognathia, posteriorly rotated ears, epicanthal folds, down slanting palpebral fissures, highly arched palate	+	ADHD	< 5%	25–50%	UR	NR	+	+	(3)
5	12q12 Deletion	+	Micrognathia, posteriorly rotated ears, epicanthal folds, down slanting palpebral fissures, highly arched palate, and full lips	+	ADHD, sensitivity	< 1%	10%	27	24	+	+	(2)
6	Y423Afs * 39	+	Down-slanted palpebral fissures with mild ptosis of outer lower lid, telecanthus, small low set ears	+	ADHD	<5%	25–50%	NR	NR	+	+	(4)
7	Deletion exons1-16	+	Thin upper lip, micrognathia and low set ears	+	Sleep disturbance	< 3%	<5%	20	19	+	+	(5)
8	Asn387*	+	Plagiocephaly, protruding ears, thin upper lip vermilion, downturned corners of the mouth	+	Anxiety	< 5%	25–50%	21	24	–	+	(5)
9	Tyr133*	+	High broad forehead with down-slanting palpebral fissures and slightly low-set posteriorly rotated ears	+	“Routine driven”	< 5%	25%	27	60	–	–	(5)
10	Gln1482*	+	Micrognathia, webbed neck	+	Rigid, anxiety	< 10%	50%	27	>60	–	–	(5)
11	Thr1564Lysfs*5	+	Thick eyebrows, ptosis, prominent eyelashes, flat nasal bridge, short nose, anteverted nares, broad philtrum, high and broad forehead and posteriorly rotated ears	+	NR	< 5%	<5%	24	>24	–	+	(5)
12	Val883Leufs*10	+	Coarse face, low anterior hairline, thick eyebrows, broad nasal tip, thick vermilion of the lower lip and micrognathia	+	Quiet	50%	<25%	12	>60	+	+	(5)
13	Deletion exons 4–21	+	Ownslanting palpebral fissures, low-set and posteriorly rotated ears, narrow and high palate, small chin	+	Anxiety	25%	<5%	NR	NR	–	–	(5)
14	Gly1139Ser fs * 20	+	Coarsening of the face, tall forehead, hypertelorism, short nose, prominent and long philtrum, thin upper lip, and full lower lip	+	NR	<3%	<25%	NR	NR	+	+	(6)
15	Arg53Glu fs * 5	+	Midface hypoplasia, horizontal palpebral fissures, and a small, upturned nose. full lower lip	+	ADHD	<3%	90%	19	NR	–	–	(6)
16	Arr 12q12-13.11 (43,005,992_ 46,669,000)	+	Epicanthal folds, down slanting palpebral fissures, webbed neck, thick eyebrows, thick upper lips, large mouth	+	–	3–5%	<3%	NR	NR	+	–	(7)

NR, no report; ADHD, attention deficit hyperactivity disorder; GERD, Gastroesophageal Reflux Disease. *Termination of translation (English Language Editors: A. Kassem and J. Chapnick).

The Chromosome test showed 46 XX, and IQ (Intelligence Quotient) test showed 80. Her bone age (BA) was 6 years old. Magnetic Resonance Imaging (MRI) of pituitary showed no abnormality (approximately 3 mm in height). There was a 2610delT frameshift mutation in the *ARID2* gene, followed by a termination codon. This mutation has not been reported previously, and it has not been recorded in in normal or disease databases. Sanger sequencing was also performed on the parents, and they were found not to carry the same mutation (Figure 1). Since this variation is a frameshift mutation (PVS1) and *de novo* variation carried by neither of the parents (PS2). It is also a variation that is absent from controls (PM2). *In silico* programs tested agree on the pathogenic prediction (PP3). It was classified as “pathogenic” according to the ACMG guidelines. Based on the results of gene sequencing and clinical features, the patient was considered as CSS6 caused by *ARID2* gene mutation.

#### Case 2

The proband (P2) was delivered at full-term *via* cesarean section (birth weight: 5.2 kg). He raised his head at 8 months, sat at 12 months, and walked at 24 months. At present, he is just over 5 years old and cannot speak, but can understand others. He is currently 105.5 cm (< 2 *SD*) in height and has a special face with dysmorphic features (including a prominent forehead, short-broad nose with anteverted nostril, thick upper lips, micrognathia, and low-set ears). Due to sudden occurrence with diabetic ketoacidosis, the patient was diagnosed with type 1 diabetes. The parents of P2 did not agree to make the child’s photo public.

**FIGURE 2:**
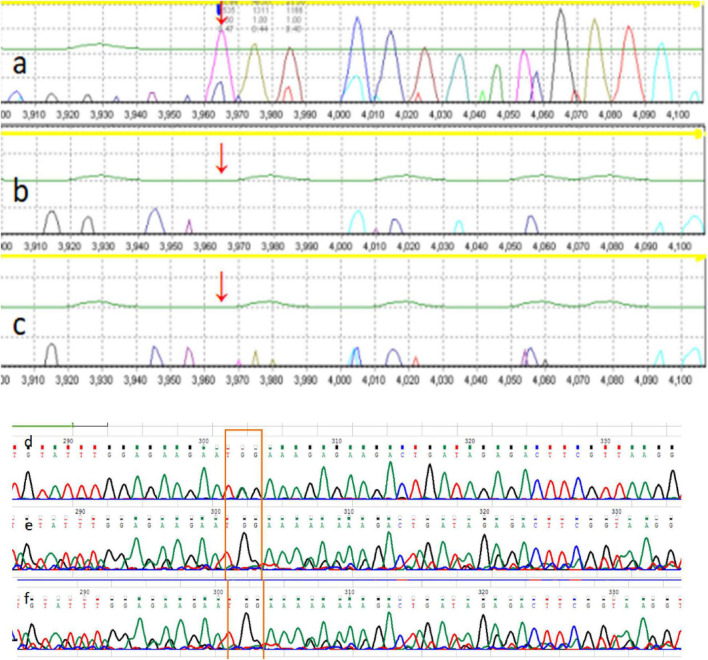
The family pedigree of patient 1. The pedigree of the family with Coffin-Siris syndrome (CSS). **(a)** P1 *ARID2* c.2610delT; **(b)** P1 father *ARID2* gene c.2610T (wild type); **(c)** P1 mother *ARID2* c.2610T (wild type). The mutation is *de novo* in the proband, whereas the parents are wild-type. The family pedigree of patient 2. The pedigree of the family with Coffin-Siris syndrome (CSS). **(d)** P2 father *ARID2* c.674G (wild type); **(e)** P2 *ARID2* c.674G > A; p. Trp225 *; **(f)** P2 mother *ARID2* c.674G > A; p. Trp225 *.

The mother also has a similar face, and her development and language were slightly delayed compared to her peers. When she was young, her learning was poor, and she often only achieved a passing grade in exams. Full exon sequencing of P2 showed that the *ARID2* gene had a nonsense mutation c.674G > A; p. Trp225 *, and Sanger sequencing showed that the mutation was inherited form the mother (Figure 2). According to the ACMG/AMP guidelines, this mutation can be identified as pathogenic. This variation is a nonsense mutation (PVS1), and it is a variation that is absent from controls (PM2). Multiple lines of evidence from *in silico* prediction indicated that the mutation was potentially deleterious (PP3). Based on the results of gene sequencing and clinical features, the child was considered CSS6 caused by the *ARID2* gene mutation.

## Discussion and Conclusion

### Clinical Features Associated With Coffin-Siris Syndrome 6

In this article, we reviewed the clinical and molecular aspects of the 16 CSS6 patients, as well as all previously reported individuals, and provided a summary of their clinical findings (Table 1). We also summarized the published phenotypes of CSS6 in the literature (Table 1, *N* = 16) (2–7). The vast majority of patients reported to date have intelligence retardation (100%) and developmental retardation (100%). These features can only be recognized during the postnatal period.

### *ARID2* Variants and Coffin-Siris Syndrome 6

Although the phenotypes of patients caused by other genes mutation in in BAF pathway have some overlap, correlation between genotype and phenotype begins to appear with the report of more individuals. ARID1B gene are the most frequently mutated in CSS. The severity of physical abnormalities and intellectual defects varies from person to person (9–12). We described two other patients with *ARID2* gene variation, which is the rarest mutated in CSS.

SWI/SNF is an ATP-dependent chromatin-remodeling complex, which controls the density and position of nucleosomes by remodeling chromatin to control the epigenetic state. It plays an important role in cell proliferation, differentiation, development, and tumor suppression. ATP-dependent chromatin remodeling is a widely used mechanism (12, 13). In mammals, 29 genes are predicted to encode protein complexes similar to yeast SWI2/SNF2. The BAF complex is evolutionarily conserved from yeast to humasns and participates in many biological processes, such as transcriptional regulation, tumorigenesis, and embryonic development. The massively parallel of sequencing technology reveals that an increasing number of somatic mutations may be to compromise in part or whole the functional activity of the SWI/SNF complex in malignant tumors. Sequencing studies have shown that 15 subunits of the BAF complex encoded by 29 genes are mutated in more than 20% of human cancers in multiple types of tumors.

BAF complex subunits vary in different tumors, and specific subunits seem to be mutated in specific cancers, highlighting the tissue-specific protective effect (14). *ARID1A* is the SWI/SNF gene with the highest frequency of tumor mutations, with mutations in more than 10% of tumors. BAF complexes can be oncogenes or tumor suppressors. Studies have shown that *ARID2* gene inactivation mutations exist in HCV (hepatitis C virus)-related liver cancer, melanoma, lung cancer, and colorectal cancer. Additionally, approximately 50% of clear cell carcinomas of the ovary and 40% of endometrial cancers have *ARID1A* mutations.

At present, there are no reports of cancer risk in CSS cases (15). A recent article on *ARID2* described the intelligence retardation and loss-of-function variants of four *ARID2* patients (3). They reported typical deformities, including frontal elevations, epicanthus epidermis, downwardly sloping eyelid fissures, micrognathia, highly arched palate, and low or posterior ears (Table 1). All patients exhibited neurodevelopmental delay, short stature, and behavioral abnormalities (including ADHD, anxiety, aggression).

Previously reported *ARID1B* gene knockout (*ARID1B* ±) mice showed neuroanatomy and expression development delays, and abnormal behaviors. Also, the *ARID1B* ± mice’s nose to buttocks length and body weight, as well as their growth hormone (GH) stimulation test indicated that *ARID1B* ± sufficient growth hormone could be secreted, but the secretion of insulin-like growth factors -1 (IGF-1) was significantly reduced (16). Celen et al. suggested that *ARID1B* ± may cause shortness due to the reduction of IGF-1 production in the brain, which leads to a decrease in plasma IGF-1. However, the inability to feed back to promote the increase of hypothalamic hormones (GHRH) and GH aggravates growth retardation. Studies have shown that the use of GH can improve growth retardation and muscle strength in mice, and the therapeutic effect has been verified in patients with *ARID1B* variants (17).

P1 exhibits the following manifestations: high forehead, short-broad nose with anteverted nostril, low-set ears, short stature, and mild intellectual disability with attention deficit and hyperactivity disorder (ADHD). GH stimulation suggests no growth hormone deficiency, and no growth hormone treatment has been given thus far. Currently, there is not enough experience in the treatment of GH in patients with short stature CSS, and most patients with CSS were treated with recombinant human growth hormone due to growth hormone deficiency (18). However, currently there is no experience with growth hormone treatment in CSS6; although children with CSS6 have short stature, GH treatment will not be administered temporarily.

As for behavioral abnormalities such as ADHD, corresponding treatment can be given. In addition, P2 did not undergo growth hormone treatment for short stature due to diabetes, and was under intellectual disability with rehabilitation therapy. Insulin was being injected subcutaneously to treat diabetes. As for the mother of P2 who has the same locus variation, intelligence and intellectual disability were not obvious.

## Conclusion

In this article, we present two cases of novel patients with CSS6 and the pathogenic variant of ARID2 and review the clinical and molecular findings of all previously reported patients. We aimed to provide a comprehensive review of the syndrome in order to help physicians to recognize the syndrome.

Overall, CSS has a spectrum of clinical manifestations. Phenotypic differences within CSS continue to be defined regarding the involved subunit of the BAF-complex or the SOX gene. Parents of P2 had no noticeable developmental or growth delays, which is inconsistent with previous findings. It appears that *ARID2* mutations in P2 are of low penetrance with no genotype-phenotype correlations. Our data challenge the current understanding that pathogenic *ARID2* mutations usually arise *de novo*.

However, considering the small sample size of the present study, further studies are necessary to determine this phenomenon. As shown in our clinical summary of 16 cases, we confirmed that CSS should be suspected postnatally in cases with global developmental delay or intellectual disability and specific dysmorphic features. The cases we reported indicate that *ARID2* mutations could have variable phenotypes, even in patients from the same family. This work can guide clinicians in performing further testing.

## Data Availability Statement

The datasets for this article are not publicly available due to concerns regarding participant/patient anonymity. Requests to access the datasets should be directed to the corresponding author.

## Ethics Statement

The studies involving human participants were reviewed and approved by the Children’s Hospital of Soochow University. Written informed consent to participate in this study was provided by the participants’ legal guardian/next of kin. Written informed consent was obtained from the individual(s), and minor(s)’ legal guardian/next of kin, for the publication of any potentially identifiable images or data included in this article.

## Author Contributions

XW, HW, and LC made substantial contributions to conception and drafted the final work. LW and HS were involved in following the patients and maintaining integrity in all parts of the work. All authors gave final approval for the article to be published.

## Conflict of Interest

The authors declare that the research was conducted in the absence of any commercial or financial relationships that could be construed as a potential conflict of interest.

## Publisher’s Note

All claims expressed in this article are solely those of the authors and do not necessarily represent those of their affiliated organizations, or those of the publisher, the editors and the reviewers. Any product that may be evaluated in this article, or claim that may be made by its manufacturer, is not guaranteed or endorsed by the publisher.

## Abbreviations

CSS: Coffin-Siris Syndrome
WES: Whole-exome Sequencing
SNVs: single-nucleotide Variants
IQ: Intelligence Quotient
BA: bone age
HCV: hepatitis C virus
GH: growth hormone
NR: no report
ADHD: attention deficit hyperactivity disorder
SGA: small for gestational age.

## References

[1] Cheng SSW Luk HM Mok MT Leung SS Lo IFM. Genotype and phenotype in 18 Chinese patients with Coffin-Siris syndrome. *Am J Med Genet A.* (2021) 185:2250–61. 10.1002/ajmg.a.62187 33768696

[2] Van PR De BP van der SS D’hondt M Fränkel U Dheedene A Confirmation of an ARID2 defect in SWI/SNF-related intellectual disability. *Am J Med Genet A.* (2017) 173:3104–8. 10.1002/ajmg.a.38407 28884947

[3] Shang L Cho MT Retterer K Folk L Humberson J Rohena L Mutations in ARID2 are associated with intellectual disabilities. *Neurogenetics.* (2015) 16:307–14. 10.1007/s10048-015-0454-0 26238514

[4] Khazanchi R Ronspies CA Smith SC Starr LJ. Patient with anomalous skin pigmentation expands the phenotype of ARID2 loss-of-function disorder, a SWI/SNF-related intellectual disability. *Am J Med Genet A.* (2019) 179:808–12. 10.1002/ajmg.a.61075 30838730

[5] Gazdagh G Blyth M Scurr I Turnpenny PD Mehta SG Armstrong R Extending the clinical and genetic spectrum of ARID2 related intellectual disability. A case series of 7 patients. *Eur J Med Genet.* (2019) 62:27–34. 10.1016/j.ejmg.2018.04.014 29698805

[6] Bramswig NC Caluseriu O Lüdecke HJ Bolduc FV Noel NC Wieland T Heterozygosity for ARID2 loss-of-function mutations in individuals with a Coffin-Siris syndrome-like phenotype. *Hum Genet.* (2017) 136:297–305. 10.1007/s00439-017-1757-z 28124119

[7] Lee Y Choi Y Seo GH Kim GH Keum C Kim YM Phenotypic and molecular spectra of patients with switch/sucrose non-fermenting complex-related intellectual disability disorders in Korea. *BMC Med Genomics.* (2021) 14:254. 10.1186/s12920-021-01104-9 34706719 PMC8555129

[8] Richards S Aziz N Bale S Bick D Das S Gastier-Foster J Standards and guidelines for the interpretation of sequence variants: a joint consensus recommendation of the American college of medical genetics and genomics and the association for molecular pathology. *Genet Med.* (2015) 17:405–24. 10.1038/gim.2015.30 25741868 PMC4544753

[9] Tsurusaki Y Okamoto N Ohashi H Kosho T Imai Y Hibi-Ko Y Mutations affecting components of the SWI/SNF complex cause Coffin-Siris syndrome. *Nat Genet.* (2012) 44:376–8. 10.1038/ng.2219 22426308

[10] Xu F Flowers S Moran E. Essential role of ARID2 protein-containing SWI/SNF complex in tissue-specific gene expression. *J Biol Chem.* (2012) 287:5033–41. 10.1074/jbc.M111.279968 22184115 PMC3281626

[11] Kang E Kang M Ju Y Lee SJ Lee YS Woo DC Association between ARID2 and RAS-MAPK pathway in intellectual disability and short stature. *J Med Genet.* (2021) 58:767–77. 10.1136/jmedgenet-2020-107111 33051312

[12] Vasko A Drivas TG Schrier Vergano SA. Genotype-phenotype correlations in 208 individuals with Coffin-Siris syndrome. *Genes (Basel).* (2021) 12:937. 10.3390/genes12060937 34205270 PMC8233770

[13] Wang Z Chen K Jia Y Chuang JC Sun X Lin YH Dual ARID1A/ARID1B loss leads to rapid carcinogenesis and disruptive redistribution of BAF complexes. *Nat Cancer.* (2020) 1:909–22. 34386776 10.1038/s43018-020-00109-0 PMC8357309

[14] Kadoch C Crabtree GR. Mammalian SWI/SNF chromatin remodeling complexes and cancer: mechanistic insights gained from human genomics. *Sci Adv.* (2015) 1:e1500447. 10.1126/sciadv.1500447 26601204 PMC4640607

[15] Van Houdt JK Nowakowska BA Sousa SB van Schaik BD Seuntjens E Avonce N Heterozygous missense mutations in SMARCA2 cause Nicolaides-Baraitser syndrome. *Nat Genet.* (2012) 44:445–9. 22366787 10.1038/ng.1105

[16] Celen C Chuang JC Luo X Nijem N Walker AK Chen F Arid1b haploinsufficient mice reveal neuropsychiatric phenotypes and reversible causes of growth impairment. *Elife.* (2017) 6:e25730. 10.7554/eLife.25730 28695822 PMC5515576

[17] Yu Y Yao R Wang L Fan Y Huang X Hirschhorn J De novo mutations in ARID1B associated with both syndromic and non-syndromic short stature. *BMC Genomics.* (2015) 16:701. 10.1186/s12864-015-1898-1 26376624 PMC4574214

[18] McCague EA Lamichhane R Holt N Schrier Vergano SA. Growth charts for individuals with Coffin-Siris syndrome. *Am J Med Genet A.* (2020) 182:2253–62. 10.1002/ajmg.a.61823 32851773

